# Environmental Noise Annoyance and Mental Health in Adults: Findings from the Cross-Sectional German Health Update (GEDA) Study 2012

**DOI:** 10.3390/ijerph13100954

**Published:** 2016-09-26

**Authors:** Friederike Hammersen, Hildegard Niemann, Jens Hoebel

**Affiliations:** 1 Institute of Social Medicine and Epidemiology, University of Luebeck, Ratzeburger Allee 160, Luebeck 23562, Germany; 2 Division of Health Reporting, Department of Epidemiology and Health Monitoring, Robert Koch Institute, General-Pape-Straße 62-66, Berlin 12101, Germany; h.niemann@rki.de; 3 Division of Social Determinants of Health, Department of Epidemiology and Health Monitoring, Robert Koch Institute, General-Pape-Straße 62-66, Berlin 12101, Germany; j.hoebel@rki.de

**Keywords:** noise, noise annoyance, transportation noise, environmental noise, ICBEN, noise pollution, mental health, MHI-5, mental disorder, environmental health

## Abstract

The health implications of environmental noise, especially cardiovascular effects, have been studied intensively. Research on associations between noise and mental health, however, has shown contradictory results. The present study examined associations between individual levels of noise annoyance due to noise from various sources in the living environment and mental health of adults in Germany. It evaluated whether these associations persisted after adjusting for potential covariates. Data were obtained from the cross-sectional “German Health Update” study 2012 (GEDA 2012), a national health interview survey among adults in Germany conducted by the Robert Koch Institute (*n* = 19,294). Noise annoyance questions referred to overall noise and that from road traffic, neighbours, and air traffic. Mental health was measured with the five-item Mental Health Inventory. Bivariate analysis showed associations between high levels of noise annoyance and impaired mental health for all noise sources except air traffic. After adjusting for covariates (sociodemographic factors, chronic disease, and social support), both men and women who reported high overall noise annoyance showed more than doubled odds of impaired mental health compared to those who were not annoyed. The odds of impaired mental health in the highest noise annoyance category from road traffic and neighbours were also significantly increased. These findings indicate that high noise annoyance is associated with impaired mental health and that this association can vary with the source of environmental noise. Further research on covariates of this association is necessary. Particularly, longitudinal data are required to establish the direction of associations and to address questions of causality.

## 1. Introduction

Environmental noise is an omnipresent environmental burden that threatens individual and public health. The World Health Organization estimates that each year more than one million Healthy Life Years are lost in the European Union member states and other Western European countries solely because of traffic noise [1]. Evidence suggests physical health risks (i.e., increased risks of cardiovascular diseases) when exposed to high levels of traffic noise [1,2,3,4]. In contrast, findings on the relation between noise and mental health are mostly inconsistent or even contradictory [5,6,7].

Mental health interacts with a wide range of complex factors, including biological, psychological, social, economic, and environmental ones [8]. The latter include not only objectively measured environmental conditions but also individuals’ subjective perceptions of their environment. This is where noise and annoyance enter the picture. Besides hearing damage caused by loud sounds, noise can evoke extra-auditory effects, such as stress reactions: For example, noise can interfere with communication, recreation, or concentration [9,10]. According to Lazarus’ transtheoretical stress model [11], stress develops when individuals perceive that their environment and associated requirements overwhelm their resources and threaten their wellbeing. If noise-induced annoyance persists (perceived as little or uncontrollable), it might cause not only stress but also fatigue associated with ineffective attempts to cope with noise. This, in turn, could impact mental health [12].

Noise can be quantified by physical parameters, such as dB(A) measured at a position around the house or in the home [3,13,14]. It can also be assessed by questions targeting noise annoyance [15]. While physical parameters measure rather objective noise exposures, questions on noise annoyance assess individual perceptions and evaluations of sounds [16]. As loud sounds are not perceived as annoying noise by everyone, answers to questions on noise annoyance provide an especially suitable indicator of the relation with mental health. Noise sources differ in their effect: Street traffic is a mostly continuous sound, while sounds from air traffic are rather intermittent. Depending on the source of noise, the same dB(A) level of noise can lead to different reported noise annoyance levels [4,17,18] and noise and noise annoyance do not necessarily correspond.

The aims of the present study were to examine the association between individual noise annoyance levels and mental health of adults in Germany and to investigate whether this association varied with the source of environmental noise and persisted after adjusting for potential covariates. The underlying cross-sectional data and theoretical background indicate the plausibility of either causal direction; however, in this analysis, we focused on the influence of environmental noise on mental health, assuming that there is a stress-related pathway by which noise annoyance affects mental health. The findings can contribute evidence on the relationships between environmental noise annoyance and individual and population health, especially for adults living in Germany, as there have been no previous studies representative for this population. Moreover, and in contrast to most previous research, this study differentiated between environmental overall noise and specific sources of noise in the living environment.

## 2. Materials and Methods

### 2.1. Study Design and Population

Data were obtained from the cross-sectional “German Health Update” (GEDA) study 2012, a national telephone health interview survey among adults living in Germany [19]. GEDA is part of the nationwide Health Monitoring System administered by the Robert Koch Institute (RKI) in Berlin [20]. The RKI is a federal institution within the portfolio of the German Federal Ministry of Health responsible for disease control and prevention. The aim of the regularly conducted GEDA surveys is to provide current data on population health, health determinants, and health service utilization for national and European health reporting systems, health policies, and public health research.

In the present study, we used data from the GEDA study 2012 (GEDA 2012), which employed a two-stage sampling procedure. First, random samples of telephone numbers from the German fixed-line network were generated using random digit dialling, which assured that households without registered telephone numbers were included in the sample. Second, the Kish Selection Grid [21]—A preassigned table of random numbers—was applied for random selection of respondents within a contacted household: The telephone interviewer recorded the number of household members, as well as their age and sex, then randomly selected the person to be interviewed according to that information. A total of 19,294 respondents aged 18–99 years completed the survey from February 2012 to March 2013. According to the internationally used Standard Definitions of outcome rates for surveys [22], the Response Rate 3 was 22.1%. This rate is the number of complete interviews divided by the number of complete and partial interviews plus the number of non-interviews and the estimated number of all eligible cases. The cooperation rate of all contacted target subjects was 76.7%. This represents the proportion of complete interviews against all contacts and refusals from known target subjects in eligible households.

Data were collected by computer-assisted telephone interviewing. The standardised interview included questions about health status, health determinants, health service utilization, prevalent diseases, and sociodemographic characteristics. The interview duration averaged approximately 1/2 hour. The study was approved by The Federal Commissioner for Data Protection and Freedom of Information, and verbal informed consent was obtained from all participants in advance of the questioning. Further information on GEDA 2012’s design, contents, survey metrics, and results can be found elsewhere [19,23].

### 2.2. Noise Annoyance

All GEDA 2012 participants were interviewed about noise annoyance in their living environment. Question wording and response scales were based on the recommendations of the International Commission on Biological Effects of Noise (ICBEN) [15]. An introductory question asked about overall degree of noise annoyance: “Thinking about the last 12 months, when you are here at home, how much does noise—all in all—bother, disturb, or annoy you”? Additional questions referred to specific sources of noise in the respondents’ living environment (road traffic, neighbours, air traffic), using the same wording as the introductory question. Participants could answer with “not at all”, “slightly”, “moderately”, “very”, or “extremely” to each question. According to the ICBEN recommendations, the response categories “very” and “extremely” were combined and labelled as “highly annoyed” (HA) [15]. The categories “slightly” and “moderately” were also combined.

### 2.3. Mental Health

Mental Health was measured with the German version of the five-item Mental Health Inventory (MHI-5), a subscale of the 36-Item Short Form Health Survey (SF-36) [24,25]. It covers anxiety, behavioural/emotional control, depression (each one item), and general positive affect (two items) [24]. The items asked about the amount of time during the past four weeks (all/most/some/a little/none of the time) in which the participants felt very nervous, so down in the dumps that nothing could cheer them up, calm and peaceful, downhearted and blue, and happy, respectively. For analysis, positively valenced items were inverted, and all items were summed into a raw score. This score was then transformed to a 0–100 point scale [26], where a value closer to 100 indicates a better mental health. Previous studies indicated good reliability of the MHI-5 (Cronbach’s α 0.74–0.84) [27,28]. A similar result was found for GEDA 2012 (Cronbach’s α 0.77). The validity of the MHI-5 has also been evaluated in previous studies. Convergent validity has been tested by comparing the MHI-5 with established instruments, and the MHI-5 proved to be comparable or superior [24,28,29,30]. The MHI-5 score is often transformed into a binary variable with reference to an external criterion, such as (not) having a mental disorder characterised by considerable mental impairments. Following the literature [30,31,32], a rather conservative cut point of 52 was chosen for this analysis: a MHI-5 score of 52 or below was classified as “impaired mental health”.

### 2.4. Covariates

Preliminary analysis of the GEDA 2012 data [33] showed that environmental noise annoyance is associated with age, socioeconomic status (SES), and urbanisation grade; thus, we considered these sociodemographic factors as potential covariates in our analysis. SES was measured with an additive multidimensional index including school/vocational education, occupational status, and net equivalent household income [34,35]. On the basis of the district typology of the German Federal Institute for Research on Building, Urban Affairs and Spatial Development, we assigned the administrative districts in which the respondents lived into three urbanisation grades: Metropolitan, urban, and rural. Social support was measured with the Oslo Three-Item Social Support Scale (OSS-3); with sum scores classified as poor (3–8), intermediate (9–11), or strong (12–14) social support [36]. Moreover, we considered a binary variable for self-reported chronic disease (yes/no) as a covariate. The distribution of each variable can be found in Table 1.

### 2.5. Statistical Analysis

In the bivariate analyses, the prevalence of impaired mental health was stratified by level of noise annoyance and source of noise. We used *p*-values derived from a Pearson’s χ²-test for a two-way table between the noise annoyance variable and the mental health variable (by noise source and sex) to examine statistically significant differences. Logistic regression models were fitted to estimate odds ratios (OR) of impaired mental health with 95% CIs and *p*-values by noise annoyance level and noise source, adjusted for potential covariates, which were added to the model step-by-step. In the first model, the ORs were adjusted for sociodemographics (i.e., age, SES, type of residential area). In the second model, chronic disease was added. The third model additionally considered social support. All analyses were conducted separately for women and men to identify sex-specific associations. Results were considered statistically significant when *p* < 0.05. We used weighting factors to account for sampling design and to adjust the sample distribution according to sex, age, education, and region to match that of the German adult population. All statistical analyses were performed using STATA 13.1 (StataCorp LP, College Station, TX, USA) survey data procedures.

### 2.6. Ethical Statement

Data were collected exclusively by computer-assisted telephone interviewing. No physical examination or laboratory testing was performed; biological samples were not collected. The study was approved by The Federal Commissioner for Data Protection and Freedom of Information in Germany (III-401/008#0015). Informed consent was obtained from all participants before the telephone interview began. Participants were informed about the goals and contents of the study, about privacy and data protection, and that their participation in the study was voluntary.

## 3. Results

Women showed a higher prevalence of impaired mental health than men (Table 1 and Table 2). In both sexes, overall noise was perceived as most annoying, followed by that from road traffic, neighbours, and air traffic, respectively (Table 2).

### 3.1. Bivariate Analysis

Across all noise sources except air traffic, increased noise annoyance corresponded with worse mental health status, indicated by a higher proportion of people with impaired mental health. In the group of women who were highly annoyed (HA) by noise overall, more than one in every four women had impaired mental health (Table 2). In contrast, impaired mental health was found in only one out of every ten women in the group not annoyed by noise overall (Table 2). Men showed the same pattern but had smaller proportions of impaired mental health throughout all noise sources and annoyance levels than women. Air traffic noise annoyance was an exception, as it was neither for women nor men significantly associated with mental health (Table 2). Although overall noise annoyance from road traffic and neighbours were each significantly associated with mental health (Table 2), in some cases, the confidence intervals of the prevalence rates overlapped between “not at all” and “slightly to moderately” annoyed. A stepwise increase in proportions of people with impaired mental health was thus only found for certain subgroups of noise annoyance level. Nevertheless, between the subgroups of HA vs. not annoyed people, differences in mental health were observed across both sexes and all noise sources except air traffic.

### 3.2. Multivariate Analysis

Multivariate analyses reinforced the results already found in the bivariate analyses, adjusted for sociodemographics (Table 3 and Table 4; Model 1): For all noise sources except air traffic, both HA men and women had significantly increased odds of impaired mental health compared with the reference category (i.e., “not annoyed”). The partial results for individual noise sources also indicated increased odds of impaired mental health for slightly-moderately annoyed vs. not annoyed respondents. The final model (Model 3), also adjusted for chronic diseases and social support, decreased the odds ratios, which lost their significance in the slightly-moderately annoyed subgroup. Nevertheless, in the final model, both men and women still had more than doubled odds of impaired mental health if HA by noise overall (Table 3 and Table 4). Raised odds ratios were also found in those reporting high annoyance by noise from road traffic and neighbours after adjustment for all covariates, with higher odds in men compared to women.

## 4. Discussion

### 4.1. Main Findings and Comparison with Previous Research

This study was among the first to examine the association between annoyance from environmental noise and mental health in adults using national data for Germany. The results demonstrate an association between high noise annoyance and impaired mental health in both men and women. The association varied according to the source of environmental noise: Overall noise annoyance showed the strongest association with impaired mental health, followed by noise annoyance caused by neighbours and road traffic (Table 2, Table 3 and Table 4). No significant associations were found between noise annoyance caused by air traffic and mental health. The adjustments for covariates attenuated the associations; however, after adjustments, HA adults still had poorer mental health than those who were not annoyed at all.

In agreement with literature findings, associations were observed between mental health and noise annoyance caused by road traffic and neighbours [37,38,39]. Additionally, as found elsewhere [38,40], these associations between noise and mental health were strongest when comparing the HA subgroup to the not annoyed one. Previous research also supported the lack of linear correlation and furthermore showed that already-vulnerable people (e.g., due to multiple morbidities) seem to be at greater risk when being highly exposed [41,42].

Our results concerning air traffic contradict those of other studies that examined noise exposure/annoyance and their connection to mental health [4,40,42,43,44,45]. We compared the results to studies using either one of the indicators, since the dependency between noise exposure caused by different sources and noise annoyance has been recorded in dose-effect curves between continuous sound level and resulting noise annoyance [4,17,18]. When the degree of noise annoyance resulting from the same noise levels was studied for different noise sources (i.e., air, road, and rail traffic), air traffic noise was found to cause the highest annoyance levels [4,18]. However, the aforementioned studies [4,40,42,43,44,45] were conducted in areas with very high levels of air traffic noise. For reasons of infrastructure, air traffic has a very uneven regional distribution [33], resulting in smaller proportions of the population being annoyed by it on a national level. Hence, in our study, the proportion of respondents HA by air traffic was relatively small; thus, the effects of this factor might be underestimated.

Striking was that the association between the highest overall noise annoyance category and impaired mental health showed the highest odds ratios after adjustment for all covariates. One possible explanation for this is that the participants might have collapsed further sources of annoyance (e.g., industrial noise or wind turbines) into overall noise. Furthermore, the interaction or sum of different noise sources might have led to the observed association. So far and to the best of our knowledge, no other study in the existing literature has examined overall noise as an isolated item. A recent study conducted in Mid-Germany found associations between high levels of noise annoyance with depression and anxiety when merging different sources of annoyance into one indicator of total noise annoyance [46].

In accordance with our postulated effective direction, the results are in line with the transactional stress model by Lazarus and Folkman [11], whereby cognitive appraisals of a stressor (e.g., noise), among other factors, leads to stress. If this stress is long-term, it might lead to impaired mental health. Honold et al. [47] found that multiple environmental burdens (e.g., noise, air pollution) often co-occur in residential areas and likely coincide with further stressors, like litter, dirt, and lack of recreational resources (e.g., urban vegetation). This demonstrates the additional necessity to integrate noise annoyance into a larger framework of environmental health determinants and develop integrated models for future research.

### 4.2. Methodological Considerations

A major strength of this study was the large sample size, with 19,294 total respondents aged 18–99 years. The sample design and weighting procedure enabled us to draw representative conclusions about the adult population in Germany. Furthermore, the use of the standardized, internationally applied ICBEN questions to assess noise annoyance allows international comparisons and future meta-analysis.

Reviews referring to inconsistent findings about noise and mental health also report associations between environmental noise and mental health problems rather than clinical psychiatric disorders [5,6,7]. By using the MHI-5 as an indicator in our study, we strove not to diagnose any particular disease pattern but capture general mental-health status.

The cross-sectional study design made it impossible to infer causality or establish the causal direction, which might be reversed from the assumed one. To clarify this, longitudinal studies are required. The data on noise annoyance, mental health, chronic disease, and social support are self-reported and thus might be influenced by confounding variables [48]. In this case, a general vulnerability that renders people susceptible to indicating elevations in both noise annoyance and mental health impairment seems plausible [49,50]. Such a general vulnerability might also include noise sensitivity. An analysis of different datasets revealed that noise sensitivity influences the development of noise annoyance: noise sensitive people tend to have a stronger correlation between the level of noise and the stated noise annoyance [49,50]. It might therefore function as a moderator. However, being highly sensitive to noise could in turn also alter the relationship between annoyance and its impact on mental health (e.g., also as a moderator). The difficulty and uncertainties of where to locate noise sensitivity are probably the reasons as to why a model that integrates noise exposure, noise sensitivity, and noise annoyance is still lacking [49,50].

The indicator showing the strongest association with mental health, the so-called annoyance caused by “noise overall”, is a global indicator that offers little potential to derive interventions from its results. Nevertheless, this global indicator may reflect a “cognitive averaging” of environmental noise from several sources, including weighting of certain sources individually perceived as more annoying than others. In turn, this may have implications for mental-health effects. Therefore, this global indicator can be considered a useful supplement to source-specific measures of noise annoyance in epidemiological studies.

## 5. Conclusions

Noise is an environmental burden whose health impacts have been the subject of numerous studies. Noise annoyance in itself can be regarded as an environmental health risk [1]; studies have found associations between high noise levels, mainly from road/air traffic, and cardiovascular diseases [1,2,3,13,51,52]. While chronic noise exposure has been shown to cause health impairments in terms of respiratory [38] and metabolic [53] diseases, findings regarding an association with mental health have been inconsistent in the past [5,6,7]. Because noise annoyance mirrors individual perceptions of sound level and disturbance [16], it may be a better indicator of noise-related burden than physical noise exposure.

To the authors’ knowledge, no representative, large-scale study has studied noise annoyance and mental health in Germany. In the present paper, we analysed data collected in the GEDA study, and the results indicate associations between high levels of noise annoyance and impaired mental health in both men and women. Hence, the results underline the importance of the living environment for mental health [54,55,56]: It determines sound exposure levels, which in turn affect noise annoyance [18].

The emergence of noise annoyance depends on factors beyond the noise level alone [57,58], for example, fear of the noise source, perceived room to manoeuvre against it [59], and individual resources to cope with or protect oneself from environmental noise exposure [60]. Furthermore, the association between noise annoyance and mental health might be influenced by further variables such as neuroticism as well as one’s frustration tolerance [61]. A larger framework considering all these factors would be fruitful, though almost impossible to create. Moreover, different environmental burdens and resources (e.g., natural environments like green areas) probably balance each other out [47]. Therefore, Honold and colleagues [47] argue that interventions providing simultaneous alterations of several environmental and psychosocial factors should be favoured over single-point-oriented strategies. Further research is necessary to determine whether an accumulation of risk factors (e.g., little psychosocial resources or deprived residential neighbourhood) corresponds with noise annoyance, how this, in turn, is related to mental health, and what role individual noise sensitivity plays in this context. An integrating framework would also be an asset in the design of longitudinal studies, which are required in the future to examine causal directionality.

## Figures and Tables

**Table 1:** Characteristics of the “German Health Update” study 2012 (GEDA 2012) study population (*n* = 19,294).

Characteristics	Women (*n* = 9976)		Men (*n* = 9318)	
	%	(*n*)	%	(*n*)
**Age**				
18–24 years	9.2	(831)	10.2	(892)
25–34 years	13.9	(1054)	15.1	(1094)
35–44 years	15.6	(1501)	17.0	(1485)
45–54 years	19.0	(1920)	20.6	(1926)
55–64 years	15.0	(1710)	15.4	(1667)
65–69 years	6.0	(760)	5.8	(627)
70+ years	21.4	(2200)	15.9	(1627)
**Socioeconomic status**				
low	23.1	(1329)	17.9	(951)
medium	59.8	(6052)	59.3	(4953)
high	17.1	(2580)	22.8	(3403)
**Type of residential area**				
rural	31.7	(3507)	32.6	(3366)
urban	40.3	(3592)	41.7	(3505)
metropolitan	28.0	(2877)	25.7	(2447)
**Chronic disease**				
yes	43.0	(4341)	38.4	(3641)
no	57.0	(5621)	61.6	(5663)
**Social support**				
poor	17.9	(1562)	16.5	(1393)
intermediate	51.2	(4933)	52.2	(4775)
strong	31.0	(3115)	31.3	(2873)
**Mental health**				
not impaired	87.8	(8792)	92.7	(8684)
impaired	12.2	(1169)	7.3	(623)
**Noise annoyance: Overall**				
not at all	56.6	(5591)	53.9	(5073)
slightly to moderately	36.8	(3707)	40.4	(3691)
highly annoyed	6.6	(671)	5.7	(549)
**Noise annoyance: Road traffic**				
not at all	62.8	(6171)	60.7	(5602)
slightly to moderately	31.8	(3233)	33.9	(3228)
highly annoyed	5.4	(569)	5.4	(485)
**Noise annoyance: Neighbours**				
not at all	68.0	(6659)	66.9	(6229)
slightly to moderately	27.8	(2915)	30.2	(2828)
highly annoyed	4.2	(397)	2.9	(258)
**Noise annoyance: Air traffic**				
not at all	80.9	(7955)	79.2	(7259)
slightly to moderately	16.7	(1747)	18.4	(1801)
highly annoyed	2.3	(267)	2.4	(256)

% = weighted percentages of respondents with complete variable information; *n* = unweighted numbers of respondents with valid information.

**Table 2:** Prevalence of impaired mental health by noise annoyance levels and noise source.

Noise Annoyance	Women		Men	
	% (95% CI)	*p*-Value	% (95% CI)	*p*-Value
**Overall**				
not at all	10.2 (9.1–11.3)	<0.001	5.9 (5.0–6.9)	<0.001
slightly to moderately	12.7 (11.4–14.2)		7.7 (6.5–9.0)	
highly annoyed	26.8 (22.0–32.1)		18.8 (14.6–23.8)	
**Road Traffic**				
not at all	11.0 (10.0–12.2)	<0.001	6.6 (5.7–7.7)	<0.001
slightly to moderately	13.4 (11.8–15.1)		7.1 (6.0–8.5)	
highly annoyed	18.8 (15.0–23.2)		16.4 (12.2–21.6)	
**Neighbours**				
not at all	10.4 (9.4–11.5)	<0.001	6.1 (5.3–7.1)	<0.001
slightly to moderately	14.8 (13.2–16.6)		8.9 (7.4–10.5)	
highly annoyed	22.7 (17.2–29.3)		18.9 (13.1–26.6)	
**Air Traffic**				
not at all	12.1 (11.1–13.1)	0.697	7.1 (6.2–7.9)	0.391
slightly to moderately	12.6 (10.7–14.8)		8.3 (6.6–10.4)	
highly annoyed	14.1 (9.3–20.9)		9.1 (4.7–17.0)	

The *p*-values were derived from Pearson’s χ²-test for a two-way table between the noise annoyance variable and the mental health variable, by sex and noise source; CI = confidence interval; % = weighted proportion of people with impaired mental health (MHI-5 ≤ 52) by noise annoyance category; MHI-5 = German version of the five-item Mental Health Inventory.

**Table 3:** Odds ratios of impaired mental health by noise annoyance levels and noise sources in women.

Noise Annoyance		Model 1 ^1^			Model 2 ^2^			Model 3 ^3^		
		OR	95% CI	*p*-Value	OR	95% CI	*p*-Value	OR	95% CI	*p*-Value
**Overall**										
	not at all (ref.)	1.00			1.00			1.00		
	slightly to moderately	1.33	(1.11–1.60)	0.002	1.28	(1.06–1.54)	0.009	1.21	(1.00–1.46)	0.047
	highly annoyed	3.04	(2.25–4.10)	<0.001	2.78	(2.05–3.78)	<0.001	2.42	(1.77–3.32)	<0.001
**Road traffic**										
	not at all (ref.)	1.00			1.00			1.00		
	slightly to moderately	1.26	(1.05–1.52)	0.014	1.20	(0.99–1.44)	0.061	1.16	(0.96–1.41)	0.131
	highly annoyed	1.77	(1.30–2.41)	<0.001	1.65	(1.19–2.28)	0.002	1.49	(1.07–2.07)	0.019
**Neighbours**										
	not at all (ref.)	1.00			1.00			1.00		
	slightly to moderately	1.58	(1.31–1.91)	<0.001	1.53	(1.26–1.85)	<0.001	1.40	(1.15–1.70)	0.001
	highly annoyed	2.42	(1.64–3.57)	<0.001	2.22	(1.51–3.26)	<0.001	1.72	(1.15–2.57)	0.008
**Air traffic**										
	not at all (ref.)	1.00			1.00			1.00		
	slightly to moderately	1.05	(0.84–1.31)	0.674	1.02	(0.81–1.28)	0.885	1.00	(0.80–1.27)	0.967
	highly annoyed	1.26	(0.78–2.03)	0.347	1.14	(0.70–1.85)	0.589	1.00	(0.62–1.62)	0.990

OR = odds ratio; CI = confidence interval; ref. = reference category; **^1^** Adjusted for age, socioeconomic status, type of residential area; **^2^** Model 1 and adjusted for health characteristics (chronic disease); **^3^** Model 2 and adjusted for psychosocial characteristics (social support).

**Table 4:** Odds ratios of impaired mental health by noise annoyance levels and noise sources in men.

Noise Annoyance		Model 1 ^1^			Model 2 ^2^			Model 3 ^3^		
		OR	95% CI	*p*-Value	OR	95% CI	*p*-Value	OR	95% CI	*p*-Value
**Overall**										
	not at all (ref.)	1.00			1.00			1.00		
	slightly to moderately	1.33	(1.03–1.72)	0.029	1.28	(0.99–1.66)	0.057	1.18	(0.91–1.52)	0.214
	highly annoyed	3.61	(2.54–5.12)	<0.001	3.31	(2.33–4.69)	0.000	2.87	(2.01–4.09)	<0.001
**Road traffic**										
	not at all (ref.)	1.00			1.00			1.00		
	slightly to moderately	1.05	(0.82–1.34)	0.704	1.02	(0.79–1.31)	0.886	0.94	(0.73–1.21)	0.643
	highly annoyed	2.48	(1.71–3.60)	<0.001	2.35	(1.63–3.37)	<0.001	2.10	(1.47–3.01)	<0.001
**Neighbours**										
	not at all (ref.)	1.00			1.00			1.00		
	slightly to moderately	1.50	(1.17–1.94)	0.002	1.43	(1.11–1.85)	0.005	1.30	(1.01–1.68)	0.044
	highly annoyed	3.18	(1.94–5.22)	<0.001	3.08	(1.89–5.02)	<0.001	2.67	(1.61–4.43)	<0.001
**Air traffic**										
	not at all (ref.)	1.00			1.00			1.00		
	slightly to moderately	1.20	(0.90–1.60)	0.208	1.13	(0.85–1.51)	0.390	1.08	(0.81–1.45)	0.596
	highly annoyed	1.42	(0.66–3.08)	0.371	1.37	(0.63–2.98)	0.434	1.29	(0.58–2.90)	0.531

OR = odds ratio; CI = confidence interval; ref. = reference category; **^1^** Adjusted for age, socio economic status, type of residential area; **^2^** Model 1 and adjusted for health characteristics (chronic disease); **^3^** Model 2 and adjusted for psychosocial characteristics (social support).

## Availability of Data and Materials

The dataset of the GEDA 2012 study supporting the conclusions of this article is available on application to interested researchers as de facto anonymized data for scientific secondary analysis. The dataset can be requested upon application from the “Health Monitoring” Research Data Centre at the Robert Koch Institute, Berlin. The data are provided on a CD-ROM. Requests should be submitted to the “Health Monitoring” Research Data Centre, Robert Koch Institute, Berlin, Germany (e-mail: fdz@rki.de). The dataset cannot be made available on a public repository because informed consent from study participants did not cover public deposition of data.

## Abbreviations

The following abbreviations are used in this manuscript:

CI: Confidence interval
GEDA 2012: German Health Update’ study 2012
HA: Highly annoyed by noise (combination of “very” and “extremely” ICBEN response categories)
ICBEN: International Commission on Biological Effects of Noise
MHI-5: Mental Health Inventory, subscale of the 36-Item Short Form Health Survey
OR: Odds ratio
OSS-3: Oslo Three-Item Social Support Scale
RKI: Robert Koch Institute
SES: Socioeconomic status
